# Improving the clinical diagnosis of familial hypercholesterolemia among patients attending diabetes and cardiology clinics

**DOI:** 10.1016/j.ajpc.2026.101431

**Published:** 2026-04-02

**Authors:** Salman M. Toor, Ralph K. Akyea, Shaban Mohammed, Moza S.H. Al Hail, Ayman El-Menyar, Mohammed Gomaa, Amin Jayyousi, Jassim M. Al Suwaidi, Nadeem Qureshi, Omar M.E. Albagha

**Affiliations:** a College of Health and Life Sciences (CHLS), Hamad Bin Khalifa University (HBKU), Qatar Foundation (QF), P.O. Box 34110, Doha, Qatar; b Qatar Biomedical Research Institute (QBRI), Hamad Bin Khalifa University (HBKU), Qatar Foundation (QF). P.O. Box 34110, Doha, Qatar; c Precision Healthcare & Drug Safety (PRISM) Research Group, Centre for Academic Primary Care, School of Medicine, University of Nottingham, Nottingham, NG7 2UH, United Kingdom; d Department of Pharmacy, Hamad Medical Corporation, P.O. Box 3050, Doha, Qatar; e Trauma and Vascular Surgery, Hamad Medical Corporation (HMC), P.O. Box 3050, Doha, Qatar; f Clinical Medicine, Weill Cornell Medicine-Qatar, P.O. Box 24144, Doha, Qatar; g Adult Cardiology, Heart Hospital, Hamad Medical Corporation (HMC), P.O. Box 3050, Doha, Qatar; h Department of Diabetes, Hamad Medical Corporation (HMC), P.O. Box 3050, Doha, Qatar

**Keywords:** Familial hypercholesterolemia, FH, LDL-C, FAMCAT, Simon Broome, DLCN

## Abstract

**Background and aims:**

Familial Hypercholesterolemia (FH) is a common autosomal dominant disorder that remains largely underdiagnosed globally (<10 %). Suspected FH cases are commonly identified using the Dutch Lipid Clinic (DLCN) and Simon Broome (SB) criteria, while the FH case ascertainment tool (FAMCAT) has been recently developed as a predictive algorithm for improving FH detection. Qatar has a high FH prevalence (1 in 125) in the general population. We determined FH prevalence and compared clinical presentation among Qatari patients with diabetes and heart disease, and between sexes.

**Methods:**

We applied the DLCN and SB criteria, and FAMCAT algorithm on data captured from a clinical cohort of native Qatari subjects (*n* = 501), recruited from two hospital-based clinics: diabetes and cardiology.

**Results:**

DLCN criteria stratified 12.6 % (95 % CI [10.0, 15.8]) as definite and 21.8 % [18.4, 25.6] as probable FH cases. SB classified 4.8 % [3.2, 7.0] as definite and 17.2 % [14.1, 20.7] as possible FH, while FAMCAT identified 35.5 % [31.5, 39.8] as suspected FH cases. DLCN showed a higher incidence of definite/probable FH in the diabetes clinic, while SB and FAMCAT identified similar proportion of definite/suspected FH cases between clinics. The three tools showed similar FH stratification between sexes.

**Conclusions:**

The prevalence of likely FH cases among diabetes and cardiology patients in Qatar was estimated at ∼1 in 3–4 patients, depending on the diagnostic criteria. Applying FH case-finding tools in hyperlipidemia patients can enhance detection rates and help mitigate FH-related morbidity and mortality through timely intervention.

**Figure:**
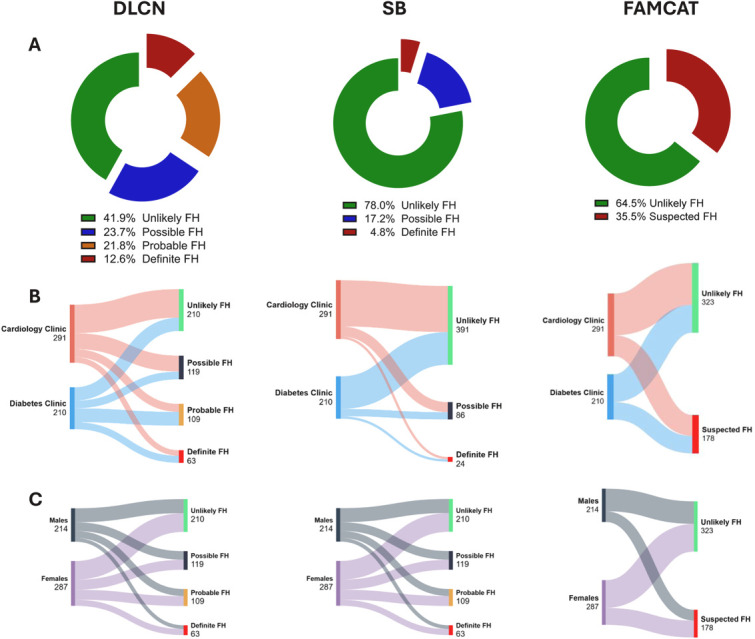
Central illustration.

## 1 Introduction

Familial Hypercholesterolemia (FH) is one of the most common autosomal dominant conditions, characterized by high levels of low-density lipoprotein cholesterol (LDL-C) in blood since birth [1]. Globally, ∼35 million people are affected by the heterozygote form of FH (HeFH) but the condition remains largely underdiagnosed with only ∼10 % clinically diagnosed cases [2]. Notably, FH prevalence greatly varies amongst different populations. FH is highly prevalent in populations with founder effect such as Ashkenazi Jews (1 in 67), Christian Lebanese (1 in 85), French Canadians (1 in 80) and South African Afrikaners (1 in 72), while a prevalence of 1 in 217 in Danish and 1 in 836 has been reported in Icelandic populations [[3], [4], [5]]. The combined global prevalence of HeFH is estimated at ∼1 in 300 in the general population, while homozygous FH (HoFH) is more severe but rare, and is estimated at ∼1 in 400,000 [6]. Untreated FH and hypercholesterolemia from birth lead to premature atherosclerotic cardiovascular disease (CVD), in particular, coronary heart disease (CHD) [[7], [8], [9], [10]].

FH is predominantly caused by genetic mutations in genes involved in LDL-C clearance [11,12]. FH diagnosis is most widely based on clinical presentation (clinical phenotype). This relates to the challenges and cost of definitive genetic testing, including feasibility and adequate coverage to detect pathogenic variants. Considering the clinical phenotype for FH, adults with FH typically present with untreated LDL-C levels much higher than the general population (>4.9 mmol/L or >190 mg/dL), a family history of elevated LDL-C levels and/or premature atherosclerotic CVD, while physical examination may indicate tendon xanthomata, corneal arcus and xanthelasmas [13]. Before assessing individuals for FH, secondary causes of hypercholesterolemia such as hypothyroidism, nephrotic syndrome, hepatic disease, and certain medications should be excluded [2]. These clinical phenotypes are broadly used to identify possible or definite FH cases in several established clinical diagnostic criteria including Simon Broome (SB) criteria [14], Dutch Lipid Clinic Criteria (DLCN) scheme [15] and the US Make Early Diagnosis to Prevent Early Death (MedPed) criteria [16], which are among the most commonly used diagnostic tools for FH. The SB and DLCN full diagnostic criteria are based on examination findings, biochemical and genetic parameters, while the MedPed criteria is based on family history of total cholesterol (LDL-C) levels beyond predefined thresholds [17]. Moreover, they have been adopted into several national guidelines and yield varying diagnostic performances among different populations [2,18,19].

More recently, a new FH case-finding algorithm, termed the Familial Hypercholesterolemia Case Ascertainment Tool (FAMCAT), was developed as a predictive algorithm to improve FH detection using routinely collected data in electronic health records (EHR). This algorithm has been developed and validated in several UK primary care datasets involving over 3 million individuals [20]. FAMCAT showed high diagnostic accuracy (AUC=0.86) in discriminating between FH cases and controls [20] and has also repeatedly shown superior discriminatory performance (AUC=0.83) and FH detection rates when compared to SB criteria and DLCN scheme [[21], [22], [23]]. FAMCAT has also been evaluated in Malaysia and the Netherlands, and outperformed both DLCN and SB criteria in terms of detection rates [24].

FH prevalence was recently estimated at 1 in 125 individuals among the general native adult population in Qatar [25], which is higher than many other countries. The Qatari population also has a high prevalence of other atherosclerotic diseases, in particular, diabetes and CVD risk factors such as obesity and hypertension [26]. Moreover, Qatar has a high rate of consanguineous marriages (∼54 %), which increases the prevalence of autosomal recessive disorders and may lead to increased homozygosity of certain autosomal dominant conditions that can lead to more severe phenotypes [27]. With FH's high prevalence, improving FH diagnosis and management is critical in reducing the risk of premature CVD in the Qatari population. Additionally, applying FH clinical diagnostic criteria on hypercholesterolemia patients have potential for improving detection rates and reducing risk of FH-related morbidity and mortality by initiation of aggressive therapy regimens.

In this study, we assessed the prevalence of clinical FH based on the DLCN, SB and FAMCAT on clinical data captured from a cohort of native Qataris, who are at increased atherosclerotic risk, from two hospital clinics. We also compared the variables that form these criteria between those recruited from the diabetes and cardiology clinics, and between men and women. Our findings enable stratification of hypercholesterolemia cases to improve FH diagnosis and management, and ultimately help tackle FH burden in Qatar.

## 2 Materials and methods

### 2.1 Study participants

The study population comprised a clinical cohort of native Qatari patients (*n* = 501) recruited from Hamad Medical Corporation (HMC, Doha, Qatar), the main healthcare provider in Qatar. Patients were recruited from two hospital-based clinics: the Cardiology clinic at the Heart Hospital (*n* = 291) and the Diabetes clinic at Hamad General Hospital (*n* = 210). The study was conducted in accordance with the Declaration of Helsinki and approved by the institutional review boards of HMC (Approval No MRC-03–20–852) and Hamad Bin Khalifa University (HBKU; Approval No 2021–03–081), Doha, Qatar. All participants provided written informed consent prior to participation in the study.

### 2.2 Study design

All study subjects provided details pertaining to clinical and family histories to fill approved questionnaires [28]. Sex classification (male/female) was based on responses in the approved questionnaire. Responses to some questionnaire-based categorical variables were missing. The missing responses were not imputed. Percentages were calculated using the full cohort as the denominator to provide consistent and conservative estimates. Patient’s clinical and biochemical measurements were also retrieved from EHR for data curation. Patients were then assessed on the DLCN and SB criteria, and FAMCAT algorithm for clinical diagnosis of FH (Supplementary Tables S1–3) [14,15,29]. The primary variables covered in FH assessment and source of retrieval are listed in Table 1. The study relied exclusively on phenotypic data only. No genetic testing for FH was performed in the present study. Lipid-lowering therapy status at highest recorded cholesterol level was used for evaluation with the three FH diagnostic tools. LDL-C correction for DLCN evaluation was performed using dose-response adjustments [30]. However, downstream comparisons using DLCN criteria were based on prevalence estimates relying on uncorrected LDL-C values to counter inflation and overestimation of FH prevalence.

**Table 1:** Source of variables included in the assessment of Familial Hypercholesterolemia using the recommended diagnosis criteria or case-finding tools.

**Variable**	**Source**	**FH assessment tool**
**Subject**		DLCN/ SB/ FAMCAT
Characteristic features	Questionnaire*	
Clinical history	EHR**	
Lipid profile	EHR	
Drug administration	EHR	
Tendon xanthoma/Arcus cornealis	Physical examination***	
**Family**		
Clinical history	Questionnaire	
Tendon xanthoma/Arcus cornealis	Questionnaire	

*Approved questionnaire. **Electronic health records. ***By Physician. DLCN: Dutch Lipid Clinic Network. SB: Simon Broome. FAMCAT: Familial hypercholesterolemia case ascertainment tool.

### 2.3 Clinical diagnosis of FH

Clinical FH was assessed using the established DLCN and SB criteria, and FAMCAT 2 tool (Supplementary Tables S1–3). Suspected FH cases were identified based on clinical and biochemical measurements. Using the DLCN criteria, patients were classified as unlikely FH (score: <3), possible FH (score: 3–5), probable FH (score: 6–8), or definite FH (score: >8). Using the SB criteria, patients were categorized as having either possible FH or definite FH, while fulfilment of set criteria defined by FAMCAT identified suspected or likely FH cases using a probability cut-off threshold corresponding to the estimated FH prevalence in the general Qatari population (1 in 125) [25]. FAMCAT assigned different weightage and coefficients to clinical variables based on sex (Supplementary Table 3).

### 2.4 Statistical analysis

The clinical characteristics of the participants included in the study were reported using descriptive measures, where frequencies (with percentages) were used for categorical variables, and median with interquartile range (IQR) or mean with standard deviation were used for continuous variables. Chi-square test was applied to determine statistical significance between categorical variables. Unpaired *t*-test was used for normally distributed continuous variables while Mann-Whitney test was applied on non-normally distributed data. A *P* value of <0.05 was considered statistically significant and represented as: *; *P* < 0.05, **; *P* < 0.01 and ***; *P* < 0.001.

## 3 Results

### 3.1 Study population

The study population comprised a clinical cohort of 501 native Qatari subjects (men; *n* = 214, 42.7 % and women; *n* = 287, 57.3 %) recruited from two hospital-based clinics (cardiology clinic; *n* = 291, 58.1 % and diabetes clinic; *n* = 210, 41.9 %). The characteristic features of the study population are presented in Table 2. The median age at the time of recruitment was 52 years, with an interquartile range of 42–61 years, and the median age at the time of highest cholesterol measurement was 50 years, ranging from 41 to 57 years. The overall mean value for the highest ever total cholesterol was recorded before treatment at 6.71 ± 1.55 mmol/l, highest ever LDL-C at 4.56 ± 1.33 mmol/l, HDL at 1.34 ± 0.36 mmol/l and triglycerides at 1.93 ± 1.39 mmol/l (Table 2). At the time of highest cholesterol measurement, 28.6 % (*n* = 143) subjects were on cholesterol-lowering therapy, compared to 63.1 % (*n* = 316) at the time of recruitment. 21.4 % (*n* = 107) reported past medical history of coronary heart disease, while 5.2 % (*n* = 26) reported history of cerebrovascular disease. Additionally, 5.2 % (*n* = 26) presented with a clinical diagnosis of chronic kidney disease (CKD), while 62.3 % (*n* = 312) presented with diabetes. Moreover, 30.7 % (*n* = 154) presented with arcus cornealis and 9.4 % (*n* = 47) presented with tendon xanthomata. A high proportion of the cohort also presented with family histories of coronary heart disease (40.9 %, *n* = 205) and hypercholesterolemia (54.5 %, *n* = 273), while no family history of confirmed FH cases was recorded in the entire study cohort.

**Table 2:** Characteristics of the study population.

	Total Cohort (*n* = 501)	Recruitment Clinic		Sex	
		**Cardiology clinic** (*n* = 291, 58.1 %)	**Diabetes clinic** (*n* = 210, 41.9 %)	**Men** (*n* = 214, 42.7 %)	**Women** (*n* = 287, 57.3 %)
**Age at recruitment** (in years), median (IQR)	52 (42 – 61)	54 (45 – 62)	50 (40 – 57)	52 (42 – 61)	52 (43 – 60)
**Ethnicity**, n (%)					
Qatari	501 (100 %)	291 (100 %)	210 (100 %)	214 (100 %)	287 (100 %)
**Cholesterol measurements** (mmol/L), mean (SD)					
Highest total chol. ever	6.71 (1.55)	6.72 (1.74)	6.70 (1.25)	6.61 (1.72)	6.79 (1.40)
Highest LDL-C ever	4.56 (1.33)	4.57 (1.56)	4.54 (0.95)	4.53 (1.52)	4.58 (1.18)
HDL at highest chol. measurement	1.34 (0.36)	1.32 (0.37)	1.38 (0.35)*	1.20 (0.3)	1.45 (0.35)*
Triglyceride at highest chol. measurement	1.93 (1.39)	1.92 (1.36)	1.95 (1.43)	2.10 (1.55)	1.81 (1.24)*
Age at highest chol. measurement	50 (41 – 57)	50 (41 – 57)	50 (41 – 57)	49 (40 – 57)	50 (42 – 57)
**Lipid-lowering therapy at highest chol.** n (%)	143 (28.6 %)	50 (17.2 %)	93 (44.3 %)*	60 (28.0 %)	83 (28.9 %)
**Lipid-lowering therapy at recruitment** n (%)	316 (63.1 %)	194 (66.7 %)	122 (58.1 %)*	141 (65.9 %)	175 (61.0 %)
**Previous history of coronary heart disease,** n (%)	107 (21.4 %)	88 (30.2 %)	19 (9.1 %)*	70 (32.7 %)	37 (12.9 %)*
**Previous history of cerebrovascular disease,** n (%)	26 (5.2 %)	22 (7.6 %)	4 (1.9 %)*	17 (7.9 %)	9 (3.1 %)*
**Family history,** n (%)					
Coronary heart disease	205 (40.9 %)	106 (36.4 %)	99 (47.1 %)*	86 (40.2 %)	119 (41.5 %)
Familial hypercholesterolaemia	0	0	0	0	0
Raised cholesterol	273 (54.5 %)	118 (40.6 %)	155 (73.8 %)*	110 (51.4 %)	163 (56.8 %)
First-degree relative with tendon xanthomata or arcus cornealis	19 (3.8 %)	15 (5.2 %)	4 (1.9 %)	7 (3.3 %)	12 (4.2 %)
**Secondary causes of hypercholesterolaemia,** n (%)					
Chronic kidney disease	26 (5.2 %)	19 (6.5 %)	7 (3.3 %)	13 (6.1 %)	13 (4.5 %)
Diabetes mellitus	312 (62.3 %)	155 (53.3 %)	157 (74.8 %)*	143 (66.8 %)	169 (58.9 %)
Physical examination**, n (%)					
Arcus cornealis	154 (30.7 %)	55 (18.9 %)	99 (47.1 %)*	60 (28.0 %)	94 (32.8 %)
Tendon xanthomata	47 (9.4 %)	7 (2.4 %)	40 (19.1 %)*	11 (5.1 %)	36 (12.5 %)*

IQR – interquartile range; n – frequency/number; SD – standard deviation; % – percentage. *Statistical significance (*P* < 0.05). **Clinical presentation at the time of data collection.

### 3.2 FH assessment using the Dutch lipid clinic network (DLCN) and Simon Broome criteria, and FAMCAT case-finding tool

**Fig. 1:**
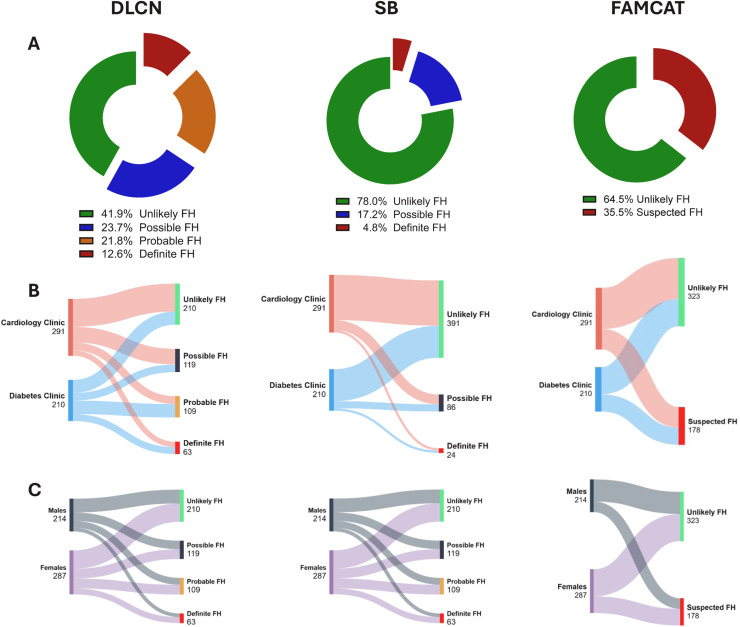
**Prevalence of clinical FH using different FH diagnostic tools and criteria.** Study participants (*n* = 501) were stratified into unlikely FH or possible/probable/definite FH cases or likely FH using the DLCN and SB criteria, and FAMCAT tool on the curated clinical data. **A.** Donut plots show the distribution of the overall stratification of study cohort into FH subtypes using the three FH diagnostic criteria. Sankey plots show the stratification of study subjects into FH subtypes using the three FH diagnostic criteria based on **B.** recruitment clinics and **C.** sex stratification. DLCN; Dutch Lipid Clinic Network, SB; Simon Broome, FAMCAT; Familial Hypercholesterolemia Case Ascertainment Tool.

The DLCN criteria (Supplementary Table 1) were used to stratify study participants into unlikely, possible, probable, or definite FH cases based on biochemical and clinical profiles (Table 2). DLCN includes past medical and family histories of coronary disease (21.4 % and 40.9 % of the study cohort, respectively), past medical history of cerebrovascular disease (5.2 %), and tendon xanthoma (9.4 %) and arcus cornealis (30.7 %) presentation. Combined, DLCN classified 63 subjects (12.6 %) as definite FH (DLCN score; >8) in the overall cohort (Fig. 1A, Table 3). 109 subjects (21.8 %) were classified as having probable FH (DLCN score, 6–8), while 119 participants (23.8 %) scored 3–5 points and were classified as having possible FH. The remaining 210 participants were classified as unlikely to have FH (DLCN score, <3). Of note, applying DLCN criteria after corrections for lipid-lowering therapy at the time of highest cholesterol measurement increased the estimates of probable/definite FH cases by 9.1 % (Supplementary Table 4).

**Table 3:** Prevalence of clinical phenotype for familial hypercholesterolemia based on the recommended diagnosis criteria or case-finding tool by the recruiting clinics and sex stratification.

	**Total Cohort** (*n* = 501)	**Recruitment Clinic**		**Sex**	
		**Diabetes clinic** (*n* = 210, 41.9 %)	**Cardiology clinic** (*n* = 291, 58.1 %)	**Men** (*n* = 214, 42.7 %)	**Women** (*n* = 287, 57.3 %)
**Dutch Lipid Network Criteria**					
Possible FH	119 (23.8 % [20.2, 27.7])	39 (18.6 % [13.9, 24.4])	80 (27.5 % [22.7, 32.9])*	55 (25.7 % [20.3, 31.9])	64 (22.3 % [17.9, 27.5])
Probable FH	109 (21.8 % [18.4, 25.6])	69 (32.9 % [26.9, 39.5])	40 (13.7 % [10.3, 18.2])*	45 (21.0 % [16.1, 27.0])	64 (22.3 % [17.9, 27.5])
Definite FH	63 (12.6 % [10.0, 15.8)	35 (16.7 % [12.2, 22.3)	28 (9.6 % [6.7, 13.6])*	24 (11.2 % [7.7, 16.1])	39 (13.6 % [10.1, 18.0])
**Simone-Broome Criteria**					
Possible FH	86 (17.2 % [14.1, 20.7])	36 (17.1 % [12.6, 22.8])	50 (17.2 %, [13.3, 21.9])	41 (19.2 % [14.4, 25.0])	45 (15.7 % [11.9, 20.3])
Definite FH	24 (4.8 % [3.2, 7.0])	12 (5.7 % [3.3, 9.7])	12 (4.1 % [2.4, 7.1])	9 (4.2 % [2.2, 7.8])	15 (5.2 % [3.2, 8.4])
**FAMCAT**					
Likely FH**	178 (35.5 % [31.5, 39.8])	81 (38.6 % [32.2, 45.3])	97 (33.3 % [28.1, 38.9])	69 (32.2 % [26.3, 38.8])	109 (38.0 % [32.6, 43.7])

Data are presented as n (% [95 % confidence interval]). *Statistical significance (*P* < 0.05). **Based on 1 in 125 prevalence [25]**.** FH; familial hypercholesterolemia. FAMCAT; Familial Hypercholesterolemia Case Ascertainment Tool.

Using the SB criteria (Supplementary Table 2) the study subjects were stratified into possible or definite FH cases based on blood lipid profiles and clinical data. 24 (4.8 %) subjects fulfilled the SB criteria for definite FH, while 86 (17.2 %) subjects were classified as possible FH (Fig. 1A, Table 3).

FAMCAT was applied using a prevalence-aligned probability cut-off, as recommended for FH case-finding [21]. A threshold of 1:125 was selected a priori to reflect the estimated prevalence of probable/definite FH in the general population of Qatar [25], in order to minimize inflation of FH probability estimates due to clinic referral bias in our high-risk clinical cohort, and to enable unbiased comparisons across the three diagnostic tools (DLCN, Simon Broome, and FAMCAT). Using this threshold, FAMCAT identified 178 participants (35.5 %%) as suspected clinical FH cases (Fig. 1A, Table 3).

### 3.3 Prevalence of clinical FH in Qatar

The DLCN criteria classified ∼13 % of the overall cohort as definite FH, while ∼22 % were classified as probable FH. Combined, ∼34 % of the entire clinical cohort were classified as probable/definite FH cases by DLCN, while FAMCAT also classified ∼35 % of the cohort as likely FH. In contrast, the SB criteria identified ∼5 % as definite FH cases and ∼17 % as possible FH, yielding a combined prevalence of possible/definite FH cases at ∼22 %. Overall, the prevalence of likely FH cases in clinics was estimated at 22–35 % or ∼1 in 3–4 patients visiting diabetes and cardiology clinics in Qatar using the three tools (Fig. 1A, Table 3).

### 3.4 Differences in FH prevalence between diabetes and cardiology clinics in Qatar

Next, we assessed the differences in prevalence of clinical FH between recruitment sites (Fig. 1B, Table 3). The number of subjects recruited from the cardiology clinic (*n* = 291) was higher than the diabetes clinic (*n* = 210). Notably, the prevalence of definite FH identified using DLCN criteria was significantly higher in the diabetes clinic (*n* = 35, 16.7 %) than in the cardiology clinic (*n* = 28, 9.6 %). Moreover, prevalence of probable FH was also higher in the diabetes clinic (*n* = 69, 32.9 %) compared to cardiology clinic (*n* = 40, 13.7 %). In contrast, prevalence of possible FH was significantly higher in the cardiology clinic (*n* = 80, 27.5 %) compared to the diabetes clinic (*n* = 39, 18.6 %). Applying the SB criteria showed a similar distribution of subjects with possible and definite FH cases from the two clinics. The distribution of likely FH cases, identified by FAMCAT was also similar in cardiology (*n* = 97, 33.3 %) and diabetes clinics (*n* = 81, 38.6 %).

### 3.5 Differences in clinical features between recruitment sites

The proportion of women (*n* = 287) exceeded men (*n* = 214) in our study cohort. Assessing differences in FH prevalence among both sexes did not show any statistically significant differences in distribution using the DLCN, SB and FAMCAT classifications (Fig. 1C, Table 3). DLCN classified 11.2 % (*n* = 24) men compared to 13.6 % (*n* = 39) women as definite FH, 21.0 % (*n* = 45) men compared to 22.3 % (*n* = 64) women as probable FH and 25.7 % (*n* = 55) men compared to 22.3 % (*n* = 64) women as possible FH. Similarly, SB criteria classified 4.2 % (*n* = 9) men versus 5.2 % women (*n* = 15) as definite FH and 19.2 % (*n* = 41) men compared to 15.7 % (*n* = 45) women as possible FH. Suspected FH cases by FAMCAT also showed similar distribution amongst likely FH cases between men (*n* = 69, 32.3 %) and women (*n* = 109, 38.0 %) (Fig. 1C, Table 3).

Comparing the clinical characteristics between recruitment sites showed some differences in clinical presentation and past medical/family histories (Table 2). Family histories of coronary heart disease were more frequent in patients from the cardiology clinic (*n* = 99, 47.1 % vs *n* = 106, 36.4 %). Moreover, while the blood lipid profiles were largely comparable between the two hospital cohorts (total cholesterol: 6.72 ± 1.74 vs 6.70 ± 1.25 mmol/l, LDL-C: 4.57 ± 1.56 vs 4.54 ± 0.95 mmol/l, HDL: 1.32 ± 0.37 vs 1.38 ± 0.35 and Triglycerides: 1.92 ± 1.36 vs 1.95 ± 1.43 mmol/l), a significantly higher proportion of participants recruited from the diabetes clinic reported family history of raised cholesterol (*n* = 155, 73.8 % vs *n* = 118, 40.6 %), use of cholesterol lowering drugs at the time of highest cholesterol measurement (*n* = 93, 44.3 % vs *n* = 50, 17.2 %), presence of arcus cornealis (*n* = 99, 47.1 % vs *n* = 55, 18.9 %) and tendon xanthomas (*n* = 40, 19.1 % vs *n* = 7, 2.4 %) compared to subjects recruited from the cardiology clinic. Subjects from the cardiology clinic showed a higher prevalence of past medical history of CHD (*n* = 88, 30.2 % vs *n* = 19, 9.1 %), cerebrovascular disease (*n* = 22, 7.6 % vs *n* = 4, 1.9 %) and CKD (*n* = 19, 6.5 % vs *n* = 7, 3.3 %) than participants from the diabetes clinic.

### 3.6 Sex-based variations in clinical presentation

The characteristic features of the cohort, stratified on differences in sex, are presented in Table 2. The median recruitment age (52 years for men and women), median age (men; 49 years vs women; 50 years) and proportion of subjects administering cholesterol-lowering drugs (men; *n* = 60, 28.0 % vs women; *n* = 83, 28.9 %) were similar among both sexes at the time of highest cholesterol measurement. The blood lipid profiles of men and women were largely comparable: total cholesterol = 6.61 ± 1.72 vs 6.79 ± 1.40 mmol/l, LDL-C= 4.53 ± 1.52 vs 4.58 ± 1.18 mmol/l but HDL-C= 1.20 ± 0.30 vs 1.45 ± 0.35 mmol/l levels were lower in men compared to women, while triglycerides = 2.10 ± 1.55 vs 1.81 ± 1.24 mmol/l levels were higher in men. Notably, past medical histories of CHD (men; *n* = 70, 32.7 % vs women; *n* = 37, 12.9 %) and cerebrovascular disease (men; *n* = 17, 7.9 % vs women; *n* = 9, 3.1 %) were higher in men compared to women. Moreover, there was a trend for higher prevalance of CKD (men; *n* = 13, 6.1 % vs women; *n* = 13, 4.5 %) and diabetes (men; *n* = 143, 66.8 % vs women; *n* = 169, 58.9 %) in men compared to women but this was not statistically significant. Arcus cornealis presentation was similar among both sexes (men; *n* = 60, 28.0 % vs women; *n* = 94, 32.8 %), while tendon xanthomata (men; *n* = 11, 5.1 % vs women; *n* = 36, 12.5 %) was higher in women than men. Family histories of CHD (men; *n* = 86, women; *n* = 119, ∼40.8 %), hypercholesterolemia (men; *n* = 110, women; *n* = 163, ∼54.1 %) and presence of tendon xanthomas or arcus cornealis in first-degree relatives (men; *n* = 7, women; *n* = 12, ∼3.8 %) were also similar among both sexes.

## 4 Discussion

### 4.1 Prevalence of clinical FH in Qatar

The FH diagnostic tools used herein yielded a prevalence of ∼22–35 % or ∼1 in 3–4 patients attending the diabetes and cardiology specialist clinics in Qatar. The prevalence of clinical FH in hospitals, particularly in patients with acute coronary syndrome, reportedly exceeds prevalence reported in the general population [[31], [32], [33]]. In agreement with these reports, the prevalence of clinical FH reported herein is higher than the general population of Qatar and reflects the enrichment of individuals with high CVD burden and marked hyperlipidemia in the clinic-based cohort. Out of 501 patients, DLCN criteria identified 63 patients (12.6 %) as definite FH cases and 109 patients (21.8 %) as probable FH. These classifications were in concordance with FAMCAT classification, which identified 178 patients (35.5 %) as likely FH, while the SB criteria identified 24 patients (4.8 %) as definite FH and 86 patients (17.2 %) as possible FH. Of note, DLCN also classified 119 patients (23.8 %) as possible FH. The remaining ∼40–80 % patients were identified as unlikely FH cases by the three tools, which further underscores the importance of utilizing FH clinical diagnostic tools for identifying high-risk patients in clinical settings, where the burden of underdiagnosed FH cases is substantial.

### 4.2 FH presentation in diabetes and cardiology clinics

As mentioned, the skewed distribution of blood lipid profile measurements towards borderline to higher levels than normal reflected the attributes of the clinical cohort. The DLCN criteria showed a higher prevalence of definite and probable FH cases in the diabetes clinic (*n* = 104, 49.6 %) than the cardiology clinic (*n* = 68, 23.4 %). In contrast, SB and FAMCAT showed similar distribution of suspected FH cases (possible/definite and Likely FH) across the two clinics. Subjects recruited from the two clinics showed similar blood lipid profiles, although HDL-C was higher in subjects from the diabetes clinic, who also showed higher administration of lipid-lowering drugs at the time of highest cholesterol measurement, perhaps due to more common initiation of preventive therapies against the development of comorbidities in diabetics. Subjects from the diabetes clinic also showed higher tendon xanthomata and arcus cornealis presentation, and stronger family histories of CHD, while subjects from the cardiology clinic presented with stronger histories of CHD and CVD. These factors contribute significantly to FH assessment by the three tools, but the effects were more pronounced in evaluation by DLCN, which yielded marked differences in FH prevalence between the two clinics.

### 4.3 FH presentation in men and women

We found a similar distribution of FH prevalence between men and women using the three tools. Although our cohort comprised a larger proportion of women, the prevalence was similar between sexes. The similar recruitment age (median of 52 years) and age at highest cholesterol measurement (49 years vs 50 years) between men and women reflect age-matched comparisons and the rigor of our findings. Some pronounced differences in clinical presentation were recorded between sexes, which included HDL-C (higher in women), triglycerides (higher in men) and a higher prevalence of CHD and CVD in men. However, tendon xanthomata were more prevalent in women compared to men. Combined, the clinical features, disease and family histories captured in FH evaluation by SB, DLCN and FAMCAT showed reciprocal contribution in the identification of suspected FH cases among men and women.

### 4.4 Relationship to literature

The American Heart Association (AHA) has defined broad clinical guidelines for FH diagnosis and proposed that FH can be clinically diagnosed based on biochemical and clinical parameters only in the absence of genetic testing, which may be supportive but is not mandatory, after exclusion of secondary causes of hypercholesterolemia such as hypothyroidism, nephrotic syndrome and obstructive liver disease [34].

The Gulf FH registry reported that FH is largely underdiagnosed and undertreated across GCC countries, and population-based studies are warranted to determine the accurate burden [35]. The Gulf FH registry study retrospectively screened 34,366 outpatient records of patients attending primary and tertiary care clinics covering cardiology, endocrine and lipid clinics in five neighboring countries: Bahrain, Kuwait, Oman, Saudi Arabia and United Arab Emirates, but it did not include Qatar. The registry cohort comprised a heterogeneous patient cohort and reported findings from the initial screening and DLCN-based classification phases, with future planned genetic testing and downstream follow-up protocols. Notably, they only relied on the DLCN criteria and estimated FH prevalence at 1:112 [35], which aligned with previous estimates in the general adult population of Qatar (1:125) [25], both higher than global estimates. In addition, the Gulf locals with acute coronary syndrome (ACS) events (Gulf COAST) registry, used a multicenter cohort of 3224 subjects hospitalized with ACS from Bahrain, Kuwait, Oman and United Arab Emirates, also reported high FH prevalence among patients with ACS, which was higher than similar European cohorts, and presented with worse prognosis than unlikely FH cases with ACS [36]. High FH prevalence among wider Arab populations has been attributed to high rates of consanguinity and endogamy, which facilitate enrichment of founder mutations [37].

Despite evidence of high regional FH prevalence, the Gulf FH Registry did not apply other FH diagnostic tools, explore population-specific diagnostic performances or targeted screening approaches to assess how FH case-finding tools can be optimized within high-risk cardiometabolic clinics, representing a significant gap in research. Overall, the Gulf FH registry provided broad surveillance for the extent of FH burden in clinical settings in the region, paving way for subsequent studies to explore and evaluate population-specific tools for targeted implementation. The Gulf COAST study reported a 3.7 % prevalence of probable/definite FH in patients with ACS [36]. Their cohort represented patients admitted across multiple Gulf hospitals with ACS, covering a spectrum of coronary artery disease (CAD) clinical presentations, and therefore representing a broad ACS/CAD cohort. The retrospective design of the Gulf FH Registry and the prospective consecutive and unselected recruitment of ACS patients in Gulf COAST study, as opposed to planned and targeted recruitment strategy in our study was expected to yield markedly higher FH prevalence estimates. Moreover, differences in referral patterns between clinics, dyslipidemia burden and systemic phenotypic characterization in our cohort may have contributed to higher FH yield recorded in our study. Targeted sampling of high-risk clinical settings enriched for severe dyslipidemia and familial risk in our study was strongly supported by consistent stratification using the three tools (DLCN, SB and FAMCAT). Combined, these reports underline the importance of focused opportunistic screening in cardio-metabolic clinics as an efficient strategy for FH identification in the region.

EHR-based FH screening tools have been widely explored for improving FH detection rates in different populations [22]. Safarova *et al.,* in the Screening Employees And Residents in the Community for Hypercholesterolemia (SEARCH) study, used an algorithm based on DLCN criteria, and reported a prevalence of 1 in 310 in the primary care setting in the US [38]. Bellows *et al.,* used clinical and genetic data of ∼50,000 individuals from the UK biobank to develop a regression model for FH variant detection and applied it to data of ∼40,000 individuals from the US National Health and Nutrition Examination Survey [39]. The authors reported that using modified DLCN for clinical criteria or genetic results alone yielded comparable results, while the combination of clinical and genetic data improved FH detection [39]. Patel *et al.,* also used EHR data of >1 million subjects (Geisinger Health System patients) and reported a FH prevalence of ∼3 % (∼1 in 33) in the overall cohort based on the modified DLCN criteria [40]. Importantly, FH was detected in ∼13.7 % or ∼1 in 7 individuals with hyperlipidemia, while adverse cardiovascular events were also significantly higher in FH cases [40]. Vickery *et al.,* also used EHR data of >150,000 Australian subjects from general practice clinics and reported that ∼1 in 5 patients showed phenotypically probable FH [41]. Combined, these studies have reported a higher prevalence of FH in a clinical setting using EHR, specifically among hyperlipidemia patients, compared to the general population. Our findings are in strong agreement with these reports and are further supported by the previously reported higher incidence of FH in the general population of Qatar [25].

Atherogenic dyslipidemias are common in both diabetic and cardiac patients [42], while hyperlipidemias can have a major influence on pre-existing diabetic and/or cardiac conditions, and can lead to increased risk of cardio-cerebrovascular events [43]. Diabetes and CHD patients are also predisposed to an increased atherosclerotic risk [44]. Therefore, using phenotypical predictors for FH detection in high-risk hospital cohorts is of crucial importance in initiating timely interventions.

Differences in lipid levels between males and females become more pronounced with age and are further augmented in the presence of dyslipidemias [45]. However, in our study, both males and females showed similar blood lipid profiles. Both SB and DLCN do not account for sex differences in FH evaluation, while FAMCAT corrects for sex differences in all predictor variables, including lipid profiles. In agreement with our findings, similar FH prevalence has been previously reported between males and females [46]. However, previously ASCVD and age of onset were lower in women who also showed less frequent administration of lipid-lowering treatments than their male counterparts affected with FH, suggesting a sex-specific impact of FH between sexes [46]. Our findings show that presentation of FH-associated clinicopathologic features, and disease burden are equally shared between both sexes in Qatar.

### 4.5 Strengths and limitations of the study

Previous studies that have applied and compared different FH diagnostic tools on EHR cohorts have been solely conducted on European or non-Middle Eastern populations. This is the first study, which reports FH prevalence in a clinical setting in Qatar and in the wider Middle Eastern region. Moreover, our study explored differences in FH detection using three different tools and clinical presentation between diabetes and CVD patients, and between men and women. Previous studies that investigated sex differences in FH have predominantly focused on differences in treatment or disease outcome as opposed to detection. Our study addresses this gap by reporting differences in clinical presentation of suspected FH cases among diabetes and cardiology patients and probed FH tool performance by sex, in an underrepresented population, adding to the diversity in available data across different populations. Lastly, the standardized collection of clinical and biochemical measurements across recruitment sites, utilization of standardized diagnostic criteria, and comprehensive assessment of family and medical histories enhance the robustness and rigor of our findings.

Despite the novelty and methodological strength of our study, some limitations warrant consideration. A significant limitation is the lack of genetic confirmation of FH in this cohort. While we applied tools based on the known high genetic prevalence in Qatar [25], we could not determine the etiology of monogenic versus polygenic hypercholesterolemia in our clinically identified cases, nor validate the tools' accuracy against a genetic gold standard covering detection of canonical FH-causing mutations. Future integration of clinical screening with genetic data is essential.

DLCN, SB and FAMCAT are inherently validated in population-based settings and may be miscalibrated when applied to specialist diabetes and cardiology clinics that are enriched for severe dyslipidemia and strong family histories, potentially inflating phenotypic FH classification. In addition, DLCN and SB comparisons in our study were based on the highest recorded LDL-C values without applying corrections for lipid-lowering therapy. This approach ensured consistent comparisons and avoided bias arising from treatment heterogeneity and treatment response, and inherent limitations in lipid-lowering correction factors, which could lead to both underestimation (due to suppressed LDL-C levels) or overestimation (due to inflated LDL-C corrected values) of definite or probable FH cases, and prioritizes identification of subjects with most pronounced FH clinical phenotype. Moreover, details on family histories of hypercholesterolemia, myocardial infarction, previously confirmed FH cases and presence of tendon xanthomata/arcus cornealis in family were self-reported, without EHR validation. Responses to some queries in the questionnaire for data capture such as those covering family histories of FH or myocardial infarction were not recorded and considered as missing. Additionally, due to lack of data on age at presentation of CHD cases in family, we were unable to classify them as premature CHD. Combined, these factors could lead to a higher estimation of probable FH cases but the clinical translation of FH screening tools favors adaptability to constraints of data availability.

### 4.6 Clinical implications

The use of structured FH clinical diagnostic criteria improves FH detection as opposed to indiscriminate testing of all patients with hypercholesterolemia. Timely identification of FH cases may be facilitated via the utilization of tools relying on biochemical and clinical phenotypical characteristics that are readily accessible via EHR. FAMCAT2 has previously shown superior feasibility than DLCN, SB and FAMCAT in terms of the incremental cost per additional monogenic FH case identified (ICER) for the identification of FH cases via cascade testing [47]. FH may be managed by a spectrum of treatments covering statins, ezetimibe and PCSK9 inhibitors, but requires timely initiation of aggressive treatment for tackling ASCVD-related morbidity and mortality [17]. We reported major secondary causes of hypercholesterolemia which are chronic kidney disease (CKD) and type 2 diabetes (T2D). These were selected a priori because of their high prevalence in our clinical population and robust documentation in the EHR. CKD is the most common comorbidity among FH patients [48], while diabetes is among the principal determinants of CVD risk among FH patients [49]. Admittance to specialist hospital clinics reiterate that urgent interventions may be required for patients at risk of early onset atherosclerotic CVD due to FH.

Although patients were selected from high CVD risk groups, the high prevalence of clinical FH in Qatar is supported by previous reports of high incidence of cardiovascular morbidities, including hypertension, dyslipidemias, and diabetes in Qatar [25,50,51]. Identification of index FH patients in the clinics can aid initiation of cascade screening protocols for identification of suspected FH cases in first- and second-degree relatives. Such an approach is greatly relevant and feasible for the Qatari population due to its modest population size and high rate of consanguinity (∼60 %) [52].

### 4.7 Future directions and research recommendations

A comprehensive approach encompassing larger patient pools from additional hospital-based clinics and more thorough data collection protocols has the potential to streamline robust FH identification in populations with higher FH rates than global estimates, ultimately reducing the disease burden. Moreover, assessment of performance of FH diagnostic criteria against genetic testing and conducting research in the wider Qatari population to identify a more generalizable approach for population screening are warranted.

## 5 Conclusion

We have utilized three tools for identification of suspected and definite FH cases in a high-risk clinical cohort from Qatar. The three tools relied on biochemical profiles, disease and family histories and clinical presentation, which are accepted criteria for initiating clinical management of FH by the AHA. Hospital-based cohorts inherently comprise a higher proportion of individuals with more advanced cardiovascular and lipid disorders. Individuals who meet the criteria for clinical FH are at elevated CVD risk and warrant initiation of aggressive intervention and management. In populations with high FH burden in the general population like Qatar, development of structured FH screening tools specifically for those attending secondary or tertiary care centers is warranted for detecting underdiagnosed FH cases. Ultimately, the clinical phenotype therefore presents a robust predictor of identifying suspected FH cases for additional evaluation and timely interventions.

## Financial support

This study was supported by a grant from the Qatar National Research Fund (QNRF) awarded to OMEA (PPM 03-0324-190038).this study.

## Data statement

All data generated or analyzed during this study are included in this published article (and its supplementary information files).

## CRediT authorship contribution statement

**Salman M. Toor:** Writing – original draft, Visualization, Methodology, Investigation, Formal analysis, Data curation. **Ralph K. Akyea:** Writing – review & editing, Investigation, Formal analysis, Data curation. **Shaban Mohammed:** Resources, Investigation. **Moza S.H. Al Hail:** Resources, Investigation. **Ayman El-Menyar:** Resources, Investigation. **Mohammed Gomaa:** Resources, Investigation. **Amin Jayyousi:** Resources, Investigation. **Jassim M. Al Suwaidi:** Writing – review & editing, Resources, Investigation, Conceptualization. **Nadeem Qureshi:** Writing – review & editing, Supervision, Investigation, Formal analysis. **Omar M.E. Albagha:** Writing – review & editing, Supervision, Investigation, Funding acquisition, Formal analysis, Conceptualization.

## Declaration of competing interest

The authors declare that they have no known competing financial interests or personal relationships that could have appeared to influence the work reported in this paper.

## Appendix Supplementary materials

Image, application 1
