# Supplementary material for: Improving the clinical diagnosis of familial hypercholesterolemia among patients attending diabetes and cardiology clinics
Source: Am J Prev Cardiol. 2026 Apr 2;27:101431. doi: 10.1016/j.ajpc.2026.101431 (PMC13261202; doi:10.1016/j.ajpc.2026.101431)
Supplement: Supplementary file 1 [file mmc1.pdf]

Supplementary Materials

Supplementary Table S1. The Dutch Lipid Clinic Network (DLCN) criteria [15] for clinical diagnosis of familial hypercholesterolaemia (FH).

**Criteria**	**Score**
**Dutch Lipid Clinic Network**	
**Family history**	
First-degree relative with premature coronary and/or vascular disease: men aged < 55 years, women aged < 60 years	1
**<u>OR</u>**	
First-degree relative with tendinous xanthomata and/or arcus cornealis	2
Children aged < 18 years with LDL cholesterol levels above the 95th percentile for age and gender	2
**Clinical history**	
Patients with premature coronary artery disease: men aged < 55 years, women aged <60yrs	2
Patients with premature cerebral or peripheral vascular disease: men aged < 55 years, women aged < 60 years	1
**Physical examination**	
Tendon xanthoma	6
Arcus cornealis, aged < 45 yrs.	4
**LDL-c concentration, mmol/l**	
≥ 8.5	8
6.5 – 8.4	5
5.0 – 6.4	3
4.0 – 4.9	1
**FH gene mutation**	
LDLR, *APOB* or *PCSK9* mutation	8
**FH diagnosis – total score**	
Definite FH	> 8
Probable FH	6 – 8
Possible FH	3 – 5
Unlikely FH	< 3

Supplementary Table S2. The Simon Broome (SB) criteria [14] for clinical diagnosis of familial hypercholesterolaemia (FH).

**Simon Broome Criteria**
**Definite FH**
Total cholesterol greater than 6.7mmol/L or low-density lipoprotein cholesterol (LDL-C) greater than 4.0mmol/L in a child aged younger than 16 years or total cholesterol greater than 7.5mmol/L or LDL-C greater than 4.9mmol/L in an adult (levels either pre-treatment or highest on treatment).
**AND**
Tendon xanthomas in patient or 1st-degree relative (parent, sibling, child) or in 2nd-degree relative (grandparent, uncle, aunt)
**OR**
DNA-based evidence of a *LDLR* mutation or familial defective apoB-100 or a *PCSK9* mutation
**Possible FH**
Total cholesterol greater than 6.7mmol/L or low-density lipoprotein cholesterol (LDL-C) greater than 4.0mmol/L in a child aged younger than 16 years or total cholesterol greater than 7.5mmol/L or LDL-C greater than 4.9mmol/L in an adult (levels either pre-treatment or highest on treatment)
**AND**
Family history of myocardial infarction: younger than 50 years of age in a 2nd-degree relative or younger than 60 in a 1st-degree relative
**OR**
Family history of raised cholesterol greater than 7.5mmol/L in adult 1st or 2nd degree relative or greater than 6.7mmol/L in child or sibling aged younger than 16 years

Supplementary Table S3. The familial hypercholesterolemia case-ascertainment tool (FAMCAT) [21,29] for clinical diagnosis of FH.

Familial hypercholesterolemia case-ascertainment tool (FAMCAT)		
**Diagnostic variables**	**Coefficients**	
	Men	Women
Highest Cholesterol measured (mmol/L)		
If LDL Cholesterol measured	0.9458	1.1927
If Total Cholesterol measured	0.5308	0.6702
Agre during cholesterol measurement (years)	-0.0297	-0.0079
Log triglycerides during cholesterol measurement (mmol/L)	-1.8518	-2.5594
Lipid lowering drugs prescribed during cholesterol measurement*		
Prescribed fibrate, bile acid sequestrant, or nicotinic acid	1.9494	1.3822
Prescribed low potency statin [a]	0.0323	1.2637
Prescribed medium potency statin [b]	0.4466	0.5440
Prescribed high potency statin [c]	0.5649	0.9350
Previous history of premature myocardial infarction*	0.8319	0.4337
Family history of familial hypercholesterolemia*	1.9076	0.9109
Family history of myocardial infarction*†	1.2621	1.4754
Family history of raised cholesterol*	1.0297	0.7521
Diagnosis of diabetes*	-1.3997	-0.7738
Diagnosis of chronic kidney disease*	-0.2528	-1.7661
Constant	-8.1134	-10.0826

*Reference group = none (b = 1); [a] Fluvastatin or pravastatin 40mg per day, or simvastatin 10mg per day; [b] Fluvastatin or pravastatin 80mg per day, or simvastatin 20 – 40mg per day, atorvastatin 10mg per day, or rosuvastatin 5mg per day; [c] Simvastatin 80mg per day, atorvastatin 20mg or more per day, or rosuvastatin 10mg or more per day; † premature defined as <55 years in men and <60 years in women.

Probability of Familial Hypercholesterolemia = $\frac{e^{xb}}{1+e^{xb}}$, where *xb* is the linear predictor of the combined coefficients and constant.

Supplementary Table S4. FH prevalence based on DLCN criteria with or without LDL-C correction for lipid-lowering therapy.

	**Without lipid-lowering therapy correction**	**With lipid-lowering therapy correction***
**Dutch Lipid Network Criteria**		
Unlikely FH	210 (41.9% [37.6, 46.2])	169 (33.7% [29.7, 38.0])
Possible FH	119 (23.8% [20.2, 27.7])	114 (22.8% [19.3, 26.6])
Probable FH	109 (21.8% [18.4, 25.6])	84 (16.8% [13.8, 20.3])
Definite FH	63 (12.6% [10.0, 15.8)	134 (26.7% [23.1, 30.8])

Data are shown as n (% [95% CI]). *LDL-C correction was performed using dose-response adjustments [30].
